# Mechanisms for nutrient interactions from organic amendments and mineral fertilizer inputs under cropping systems: a review

**DOI:** 10.7717/peerj.15135

**Published:** 2023-04-04

**Authors:** Benedicta Essel Ayamba, Robert Clement Abaidoo, Andrews Opoku, Nana Ewusi-Mensah

**Affiliations:** 1 Soil Fertility and Plant Nutrition Division, CSIR-Soil Research Institute, Kwadaso, Kumasi, Ghana; 2 Department of Crop and Soil Sciences, Faculty of Agriculture, Kwame Nkrumah University of Science and Technology (KNUST), Kumasi, Ghana; 3 Department of Theoretical and Applied Biology, Kwame Nkrumah University of Science and Technology (KNUST), Kumasi, Ghana; 4 International Institute of Tropical Agriculture (IITA), Oyo Road, Ibadan, Nigeria

**Keywords:** Food production, Mechanism, Nutrient priming effect, Nutrient synchrony, Soil fertility improvement

## Abstract

Food security issues continue to be a challenge in most parts of the globe, especially in sub-Saharan Africa (SSA). Several research attempts on addressing this issue have mainly been on nutrient replenishment using combined nutrient application of organic amendments and mineral fertilizer inputs. However, there is limited information available on the potential mechanisms underlying nutrient interactions associated with co-application of organic amendments and mineral fertilizers. Therefore, this review focuses on the mechanisms underlying crop nutrient interactions, with particular emphasis on improved nutrient synchrony, priming effect, general soil fertility improvement and balanced proportion of nutrients required by crops. Following a brief overview of the mechanisms, the review describes four common pre-determined nutrient ratios required by plants depending on its life cycle, environment and genotypic characteristics in order to attain the crop’s maximum genetic potential. The review concludes with the need for future research to understudy mechanisms causing nutrient interaction under cropping systems, so as to apply nutrients at the most appropriate time to synchronize nutrient release with crop uptake, with the utmost goal of promoting sustainable crop production and enhancing food security.

## Introduction

Declining soil fertility with its consequent suboptimal crop yields has been a major crop production constraint in diverse agro-ecologies over the years (Sanchez, 2002; Ayamba et al., 2021). Due to this, most of current research have focused on replenishing soil fertility using combined application of organic amendments and mineral fertilizer inputs as well as developing strategies to mitigate climate change effects on sustainable crop production. The proponent of this approach have it as meaningful for achieving the global sustainable development goals (SDGs) 1 and 2 (Blesh et al., 2019; Khanal et al., 2021). Specifically, the approach targets the outcome of ending hunger and improving access to food, achieving food security and improved nutrition, and promoting sustainable agriculture and resilient agricultural practices in sub-Saharan Africa (SSA).

Nutrient interaction in crops is a major factor influencing the yields of annual crops (Rietra et al., 2017). They occur in crop plants when the supply of one nutrient influences the absorption and utilization of other nutrients (Rietra et al., 2017). These interactions are classified as either synergistic, or additive. A few studies have reported positive nutrient interactions/added benefits from combined nutrient application of organic amendments and mineral fertilizer inputs to crops, ranging from 118 to 663 kg ha^−1^ extra crop yields (Nhamo, 2001; Mucheru et al., 2004) with over 400% grain yield increases over the control (Chivenge et al., 2009).

While most of the studies highlighted the positive impacts of combining nutrient inputs on crop yields, few studies quantified the extra crop yields attained, to predict whether the additional yields were the result of synergistic, antagonistic or additive interactions. Despite divergent opinions and ideologies on nutrient interaction, the actual underlying mechanisms involved are not well understood. Scotti et al. (2015) opined that the positive effect of nutrient amendments on crop yields are attributed to different mechanisms, and thus, not mutually exclusive. Possible mechanisms for added benefits/disadvantages in grain yields resulting in synergistic/antagonistic interactions have been attributed to improved nutrient synchrony between crop nutrient demand and soil nutrient release (Vanlauwe, Wendt & Diels, 2001a; Crews & Peoples, 2005); priming (Kuzyakov, Friedel & Stahr, 2000; Kuzyakov, 2010); improvement in soil quality indicators (Vanlauwe et al., 2001b; Rietra et al., 2017; Sanginga & Woomer, 2009); preferential transport of nutrients (Rietra et al., 2017); balanced nutrient ratios (Essel et al., 2021), *etc*. However, there is limited information on the potential mechanisms causing added benefits from the application of both organic amendments and mineral fertilizers. There is therefore an urgent need to quantify the contribution of these mechanisms to added benefits in grain yields. This is necessary because knowledge of the exact mechanism causing nutrient interactions will enhance the adoption of soil management practices that will contribute to improvement in crop productivity and soil fertility.

The aforementioned gap in knowledge formed the basis of this review. The objective of this review was therefore to investigate the following mechanisms: improved nutrient synchrony, priming effect, general soil fertility improvement and balanced proportion of nutrients required by crops that influence nutrient interactions from organic amendments and mineral fertilizer inputs under cropping systems, with the aim of enhancing a sustainable food production system and mitigate against food insecurity in Africa.

## Search Methodology

This review began with the formulation of research questions and hypothesis, after which the scope was defined. The formulated research hypothesis was that “the nutrient interactions resulting from combined application of organic amendments and mineral fertilizers to crops are influenced by mechanisms such as nutrient priming, nutrient synchrony, soil fertility and balanced ratio of nutrients”. To ensure a rigorous investigation of literature so as to test the hypothesis and achieve the objectives of this review, a comprehensive investigation of published research on “mechanisms for nutrient interactions from organic amendments and mineral fertilizer inputs under cropping systems” was employed, following the approach by Khan et al. (2003). We conducted a literature search using Google scholar (scholar.google.com) and Scopus (Elsevier) literature database (https://www.scopus.com). Keywords and phrases such as “mechanisms for crop nutrient interactions”, “nutrient priming effect”, “nutrient synchrony”, “soil fertility improvement”, and “soil nutrient ratios” were used. Related articles were then extracted to categorize and summarize the underlying mechanisms causing nutrient interactions from organic amendments and mineral fertilizer inputs under cropping systems. The search was restricted to references that included plant science and agronomy.

## Review

### Mechanisms for nutrient interactions

Several reasons have been attributed to the extra crop yields obtained from combined application of organic inputs and mineral fertilizers. Vanlauwe, Wendt & Diels (2001a) made an assertion that, an extra resultant yield increase from the nutrient inputs was due to an improvement in synchrony between the crop nutrient demand and soil nutrient release. In another experiment carried out in the same year, Vanlauwe et al. (2001b) reported that, extra crop yields were due to the ability of the manure to improve soil quality indicators such as cation exchange capacity and pH for the effective utilization of mineral fertilizer nutrients. However, without a clear understanding of the mechanisms underlying the enhanced crop yields resulting from synergistic interactions of the combined application of mineral fertilizer and organic inputs, one can underestimate the occurrence of antagonistic interaction (Vanlauwe & Sanginga, 2004).

Rietra et al. (2017) attempted to relate important nutrient interactions to the preferential transport of nutrients, effects of nutrients on phytosiderophore production and reductase activity, and management strategies. Many other authors have reported that some synergistic interactions resulting from nutrient application may be because of the soil pH being influenced by the acidifying effects of fertilizer application. Kuzyakov, Friedel & Stahr (2000) also reported that predation, nutrient competition between roots and microorganisms, preferred uptake are some of the mechanisms influencing nutrient interactions. Nhamo (2001) also attributed added benefits in grain yields of maize resulting from application of cattle manure and ammonium nitrate fertilizers to the release of basic cations from cattle manure. According to the authors, the basic cations released from the cattle manure may have alleviated the constraints associated with crop growth caused by the low cation exchange capacity (1.20–2.50 cmol _(+)_/kg) of the sandy soils.

However, many researchers may have diverse opinions and ideologies regarding the mechanisms underlying nutrient interactions. This is because mechanisms involving soil and plant nutrient interactions are not well understood. Studies on integrated nutrient management have reported improvement in crop yields; however, the added benefits in yields are rarely quantified and the actual mechanisms underlying these interaction effects are not clearly known (Badu, 2014). There is therefore an urgent need to generate a detailed understanding into the potential mechanisms for added benefits from the combined application of both organic amendments and mineral fertilizers (Vanlauwe et al., 2002). Although some may consider this as an illusion, due to the nutrient losses associated with the field application of nutrients, incubation experiments can be carried out under controlled conditions to understand the potential mechanisms for nutrient interactions. In a study conducted by Badu (2014) on assessing the relative contribution of key mechanisms to the synergistic interactions resulting from the combined application of manure and mineral fertilizers, the author reported that, the key mechanisms resulting in synergistic interactions were general soil fertility improvement, priming effect and improved nitrogen synchrony. Essel et al. (2021), in a similar study, also reported that priming effect, balanced nutrient ratio, and nutrient synchrony cumulatively explained 86 percent of the variation among the mechanisms contributing to added benefits which resulted from combined application of compost and mineral fertilizer in a maize cropping system.

### Improved nutrient synchrony

The concept of nutrient synchrony refers to a balance between nutrient supply and crop demand. It serves as a linkage between nutrient release from mineralization of nutrient inputs with the crop nutrient requirements (demand) (Crews & Peoples, 2005). Tetteh (2004) explained synchrony as a means of manipulating nutrient inputs to enable nutrient release and crop uptake to occur concurrently and improving the efficiency of nutrient uptake by crop plants. The concept of nutrient synchrony has been applied in various aspects of traditional farming systems. Some examples are, the widely practiced split-application of nitrogen fertilizers (Deligios et al., 2021), and also the planning of sowing times at the beginning of the rainy seasons to enable crops make maximum use of moisture from the rains and the flush of soil nutrients occurring after the first few rains in the growing season (Tetteh, 2004). For instance, the maize crop has specific nutrient requirements at the different phenological stages of crop growth as presented in Table 1. Hence, for nutrient synchrony to occur, nutrient application is usually done at a time that will synchronize with the crop demand, especially at the critical stages of crop growth, notably at tasselling and grain filling. Nutrient uptake by maize has been reported to be minimal in the early phases of plant development, and rapidly increased to a maximum before and after tasselling (International Fertilizer Industry Association (IFA), 1992).

**Table 1:** Pattern of nutrient uptake by maize at different phenological stages of crop growth.

Phenological stages	Days after sowing	Nitrogen uptake (kg/ha)	Phosphorus uptake (kg/ha)
V_E_ to V_1_ (seedling)	0–14	19	2
V_2_–V_n_ (Rapid vegetative growth)	15–47	84	12
V_T_–R_1_ (Flowering and fertilization)	48–56	75	16
R_2_–R_5_ (Grain filling)	57–90	48	11
R_6_ (Maturity)	91 -95	14	3

**Notes.**

Source: Johnston and Dowbenko (2004).

Nutrient synchrony by crops is critical to reduce inefficient nutrient use and nutrient loss by volatilization and leaching (International Fertilizer Industry Association (IFA), 1992). Furthermore, when crop demands for nutrients are closely synchronized with processes that regulate the availability of nutrients in soils, acidification of soils is minimized (Crews & Peoples, 2004). Deligios et al. (2017), in their study on globe artichoke, incorporated cover crops into the soil a few weeks before planting to better synchronize nutrient release from the cover crop residue as a result of decomposition and nutrient uptake by the subsequent crop planted. In regard to nitrogen and phosphorus synchrony, Tetteh (2004) reported that nitrogen synchrony can be attained due to the different conversion forms of nitrogen depending on management practices carried out. Similarly, availability and mobility of phosphorus in soils can be manipulated to attain synchrony with crop demand (Tetteh, 2004).

Some simple management practices that can be adopted to improve nutrient synchrony are: changing the time of N application during the cropping season as well as the type of fertilizer used, practicing band placement of fertilizer, and practicing split-N application (Crews & Peoples, 2005; Deligios et al., 2021).

### Priming effect

Priming effect is a fundamental phenomenon occurring in most natural ecosystems. It is known as one of the most important but poorly understood phenomenon influencing mineralization of soil organic carbon (Cardinael et al., 2015). Kuzyakov, Friedel & Stahr (2000) defined priming effect as “the strong short-term changes in soil organic matter turnover induced by relatively moderate treatments of the soil”. According to the authors, large amounts of C, N and other nutrient elements can either be mineralized (positive priming) or immobilized (negative priming) in the soil within a very short period of time. Priming effect is a phenomenon used to explain the changes in soil organic matter (SOM) decomposition due to the modifications SOM does in the pool composition (Kuzyakov, 2010). There have been diverse approaches in the explanation of priming effect with respect to either carbon or nitrogen as nutrient sources. Most studies on C priming, defined priming effect as an extra decomposition of organic C after the addition of easily–decomposable organic substances to the soil (Kuzyakov, Friedel & Stahr, 2000). For instance, Ji et al. (2018) and Wu et al. (2019) observed that long-term fertilization regulated the intensity of SOM priming and carbon balance after organic fertilizer addition. The priming effect of most N priming research was described as the additional soil N taken up by crops after addition of nitrogen fertilizer, compared to the control plots (Kuzyakov, Friedel & Stahr, 2000).

In order to evaluate priming effect, the carbon input in a soil is compared to a control soil where no substrate was added. Kuzyakov (2010) has suggested that incubation experiments are needed to quantify this mechanism due to the way it simulates the behaviour of SOM inputs in natural ecosystems. Priming effects occur mainly in the rhizosphere of plants. The duration of occurrence of priming effects in soils is very crucial as most priming experiments are usually conducted over a short period of time, lasting for about three months.

**Figure 1:**
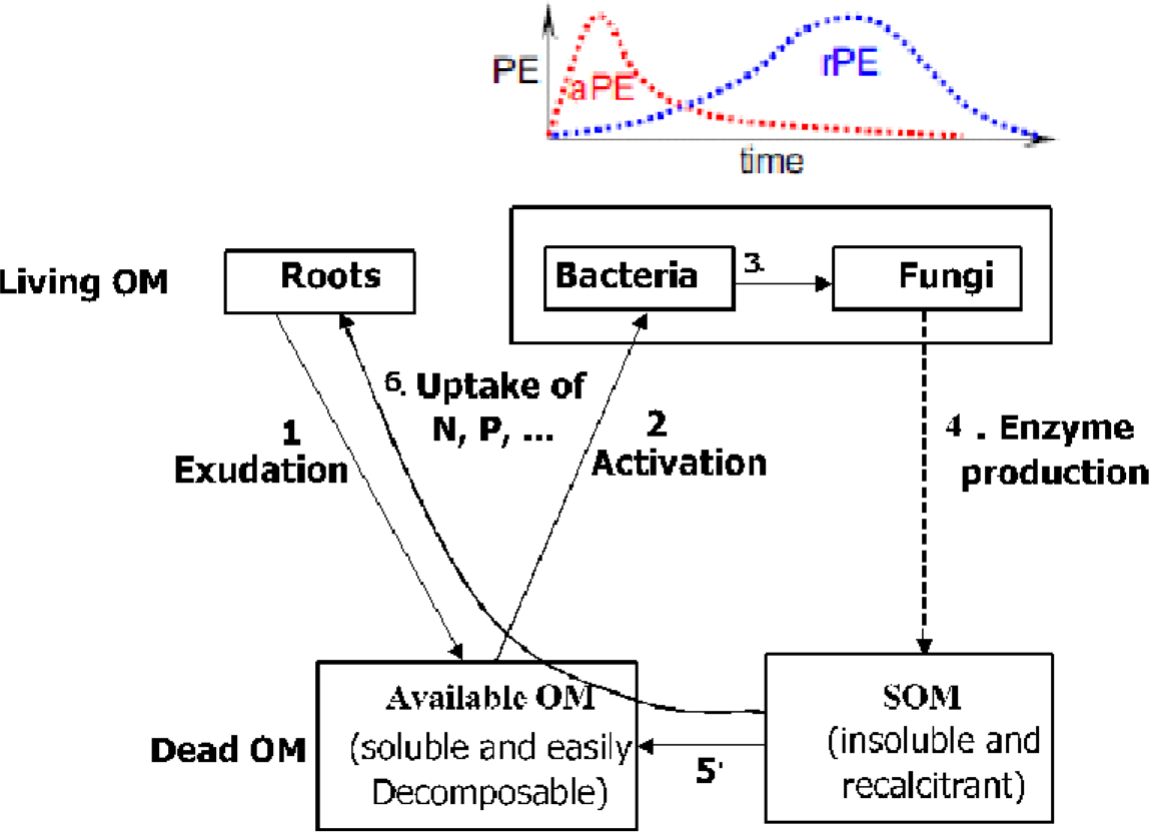
Sequence of processes inducing real (rPE) and apparent (aPE) priming effects (Kuzyakov, 2010).

Two types of priming effects have been identified from a review by Kuzyakov (2010); apparent priming and real priming effects. Although Kuzyakov (2010) reported that nutrient priming occurs immediately after the addition of substrates, it has been found that “real” priming may be staggered for several days or weeks after substrate addition. Whiles the apparent priming occurs immediately after substrate addition to increase the SOM turnover, real priming delays for several days or weeks. The sequence of processes influencing priming effects are presented in Fig. 1.

Real and apparent priming effects can either be negative or positive, depending on the type of substrate added to the soil. Substrates with C:N ratio greater than 10 leads to negative priming effects, whereas those with C:N ratio less than eight results in positive N priming effect (Kuzyakov, Friedel & Stahr, 2000), as later shown by Barłóg, Hlisnikovský & Kunzová (2020) who reported that a high rate of mineral fertilizer inhibits priming effect. In view of this, Kuzyakov, Friedel & Stahr (2000) summarized the potential causes of the different priming effects, arising from the addition of C and N inputs and these have been presented in Table 2.

**Table 2:** Potential causes of priming effects, resulting from the addition of C and N inputs.

Target	Priming effect	Potential causes
Carbon	Positive real priming effect	Acceleration of SOM mineralization as a substrate and energy source and N immobilization through an increase in microbial activity
	Negative real priming effect	Reduction in C:N ratio
		Microbial immobilization of C
		Microbial immobilization of N due to readily-available C-rich substrate in the soil
Nitrogen	Positive real priming effect	Increase in atmospheric N_2_-fixation
	Negative real priming effect	Decrease in atmospheric N_2_-fixation Microbial immobilization of N
	Positive apparent priming effect	Stimulation of N uptake by roots
	Negative apparent priming effect	NH_4_^+^ fixation by clay minerals
Carbon and nitrogen	Positive real priming effect	Increase mineralization of SOM through a lower C:N ratio
		Increase in microbial activity and facilitation of SOM mineralization by means of co-metabolism
	Negative real priming effect	Switching of microbial biomass from soil organic matter on the easily available C and N sources
		Preferred uptake of C-rich substrates by microorganisms
	Negative apparent priming effect	Incomplete decomposition of C and N sources
		Sorption of physico-chemical protection and immobilization of added substrates

**Notes.**

Source: Adapted from Kuzyakov, Friedel & Stahr (2000).

### General soil fertility improvement

One of the mechanisms known to cause nutrient interactions after the conjoint application of organic and inorganic nutrient amendments is general soil fertility improvement. It was reported by Sanginga & Woomer (2009) that the supply of essential nutrient elements in adequate proportions enhances general soil fertility improvement which is a possible mechanism for synergistic interactions. The combined application of organic and inorganic fertilizers contributes to soil fertility and productivity of crops through its positive impact on the physical, biological and chemical properties of the soil, especially on a long-term basis (Liza et al., 2014). For example, Vanlauwe et al. (2001b) noted that additional crop yields arising from the combined use of nutrient inputs were due to the manure’s ability to enhance soil quality indicators viz cation exchange capacity and pH for the efficient use of mineral fertilizer nutrients. Also, acidifying effects of mineral fertilizer can influence soil pH, and lead to synergistic interactions (Rietra et al., 2017). While inorganic fertilizers are known to supply adequate levels of macro nutrients, the organic fertilizer supplies micro nutrients which otherwise are absent in the inorganic fertilizer (Sanginga & Woomer, 2009). This mechanism often results after nutrient release synchronizes with crop demand causing mineral fertilizer to readily release nutrients for crop uptake whiles the organic fertilizer staggers the release of nutrients to a later stage in the growth of crops (Palm, Myers & Nandwa, 1997; Vanlauwe, Wendt & Diels, 2001a). For instance, the combined application of cattle manure and mineral fertilizer resulted in added benefits in the grain yield of maize due to soil fertility improvement caused by the release of basic cations from cattle manure (Nhamo, 2001). Badu (2014) reported general fertility improvement mechanism represented by N and P release as the least contributing mechanism (11.15%) to synergy resulting from the conjoint application of manure and mineral fertilizer.

### Balanced ratio/proportion of nutrients

Crops require essential nutrients for their growth and yield. In order for crops to attain optimum growth and yield, there is the need to supply adequate nutrients in the proper amounts and balance to the crops. There are 16 essential nutrient elements required for the proper growth and development of crops. With the exception of carbon, hydrogen and oxygen which are derived from the atmosphere, the remaining 13 are derived solely from the soil if no nutrient amendments are applied. Out of these, 83% of the total nutrients absorbed constitute N, P and K; 16% is absorbed by the secondary nutrient elements (Ca, Mg and S); and the remaining 1% is absorbed by the micronutrients (Cl, Cu, B, Fe, Mn, Zn and Mo) (Corn Growers’ Workshop, 2018).

Several researchers have suggested balanced nutrient application using NPK fertilizers as a key strategy for enhancing crop productivity (Agyin-Birikorang et al., 2022; Wang et al., 2021); however, some farmers have resorted to imbalanced fertilization (that is the use of one fertilizer for their crops, for example maize and rice) (Yousaf et al., 2017). Balanced fertilization of nutrients is an efficient way of minimizing nutrient losses whiles improving the nutrient use efficiency of crops. The balanced fertilization of NPK produced the highest yields in rice-oilseed rape rotation in China. According to the aforementioned authors, the increased yields were as a consequence of the balanced supply of all essential nutrients required by the plants. The other treatments used by the authors which lacked at least one essential nutrient, recorded low yields due to the specific nutrient deficiency and stress induced by the imbalanced fertilization. Linear increases in grain yields can result when nutrients are taken up in balanced proportions, until the yield attained is about 60–70% of the yield potential of the crop.

Maize plants have a relatively high demand for nutrients, especially, nitrogen, phosphorus and potassium in order to produce high yields. A maize variety with a yield potential of 5–6 Mg/ha will have a nutrient uptake of 100–150 kg/ha N, 40–60 kg/ha P_2_O_5_ and 100–150 kg/ha K_2_O. However, most soils cannot supply more than 20–25% of the NPK requirements of the crop (Maize Production Manual, 1982; Aliyu et al., 2021). Due to the low fertility of most soils, there is an urgent need for balanced nutrition of maize. Rietra et al. (2017) made a report that nutrient deficiency or imbalance is one of the primary causes of low crop yields and as such it is imperative to apply balanced amounts of nutrients in the soil. This can be achieved by considering the essential nutrient elements and fine-tuning them to local soil chemical conditions and crop requirements.

Oenema & Velthof (2002) documented that application of balanced proportion of nutrients is an ambiguous term and has several definitions ranging from mutual harmony that results when the availability of all essential nutrients is adjusted to crop demand to the steadiness that results from nutrient uptake/removal through harvest. Mahajan & Gupta (2009) also emphasized that balanced fertilization does not only refer to the application of a definite proportion of nutrients in the form of mineral fertilizers to the soil, but also includes the use of organic amendments. Graeme Sait defined nutrient ratios in simple terms as important ratios required to promote soil health and crop production. The popularly known Mulder’s Chart also highlighted some nutrient interactions occurring among nutrient elements. From Mulder’s Chart, plant nutrients interact with each other, with the presence of one nutrient element influencing the availability, uptake, distribution and proper functioning of another nutrient element either positively (stimulation) or negatively (antagonism) (Lai et al., 2019). For example, high nitrogen levels decrease the availability of potassium, boron and copper, whiles increasing the demand for magnesium. High phosphorus levels can reduce calcium, iron, potassium, copper and zinc uptake. High potassium levels in soils can reduce the availability of magnesium, whiles positively stimulating manganese uptake. Due to the interactions resulting from nutrient elements, balanced ratios of nutrients are more critical than the actual concentration of single nutrient elements (Malvi, 2011).

The concept of applying balanced proportion of nutrients is well understood, however its implementation is hindered by inadequate practical guidelines for the verification of this mechanism. Estimation of nutrient balances in farmlands is vital in determining whether the application of balanced proportion of nutrients is beneficial. However, due to the nutrient losses associated with the field application of nutrients, laboratory incubation experiments can be carried out to quantify this mechanism. According to Malvi (2011), there is a pre-determined ratio of nutrients required by plants depending on its life cycle, environment and genotypic characteristics to attain the crop’s maximum genetic potential. Due to this, many nutrient ratios have been identified. These are: Ca: Mg (3: 1), K: Mg (1:1), P: S (1: 1), P: Zn (10:1) and Fe: Mn (2:1) ratios.

### Calcium to magnesium ratio (3:1)

This is the most important ratio in the soil. It determines the gaseous exchange in soils for better photosynthesis in crops. Ca: Mg ratios are needed in balanced proportion because a high magnesium concentration in soils may inhibit the activity of aerobic microorganisms in the soil (Sait, 2015a). The Ca: Mg ratios recommended for sandy soils is 3: 1 and clayey soils is 7:1 (Philips, 2021; Osemwota, Omueti & Ogboghodo, 2007).

### Potassium to magnesium ratio (1:1)

This is the second most important ratio. The presence of one nutrient element may influence the uptake of another element either negatively or positively. The presence of high magnesium in the soil, inhibits the uptake of potassium and vice versa, leading to poor yields. However, a balanced ratio will enhance the proper growth and development of the crop (Järvan, 2004).

### Phosphorus to sulphur ratio (1:1)

This ratio takes into account an often neglected mineral element; Sulphur. Three decades ago, sulphur was mainly supplied through rains. However, after it was realized most of the acid rains were from industrial operations, resulting in dying waterways and forests around the globe, most soils have been reported to be sulphur deficient. Thus, there is the need to optimize P:S ratio for proper crop nutrition (Essel et al., 2021).

### Phosphorus to zinc ratio (10:1)

It is very important to supply zinc and phosphorus in a ratio that ensures maximum performance of both nutrient elements. High phosphorus levels in soils can inhibit zinc uptake and result in poor yields of crops. The ideal P: Zn recommended ratio for crops is 10: 1 (Essel et al., 2021; Philips, 2021).

### Iron to manganese ratio (2:1)

Iron and manganese are essential micronutrients for plant resilience. Iron and manganese are antagonistic to each other, hence excess manganese in the soil could lead to iron deficiency (Sait, 2015b; Essel et al., 2021).

## Conclusions

The mechanisms for nutrient interactions from organic amendments and mineral fertilizer inputs under cropping systems were reviewed in this article. While most of the studies highlighted the positive impacts of combining nutrient inputs on crop yields, few studies quantified the extra crop yields attained in order to predict whether the additional yields were the result of synergistic, antagonistic or additive interactions. This resulted in a subsequent review of four potential mechanisms considered to cause added benefit, thus priming effect, nutrient synchrony, soil fertility improvement and balanced ratio of nutrients. As envisioned by the United Nation’s Agenda 2030 to achieve a better and more sustainable future for all people and the world by 2030, this review recommends the need to understand mechanisms underlying nutrient interactions under cropping systems so as to apply nutrients at the most appropriate time to synchronize nutrient release with crop uptake. It is believed this will promote sustainable crop production and enhance food security.

##  Supplemental Information

10.7717/peerj.15135/supp-1 Supplemental Information 1 Search methodology

## Additional Information and Declarations

### Data Availability

The following information was supplied regarding data availability: This is a review article.

## References

[ref-1] Agyin-Birikorang S, Adu-Gyamfi R, Tindjina I, Fugice J, Dauda HW, Sanabria J (2022). Synergistic effects of liming and balanced fertilization on maize productivity in acid soils of the Guinea Savanna agroecological zone of Northern Ghana. Journal of Plant Nutrition.

[ref-2] Aliyu KT, Huising J, Jibrin JM, Mohammed IB, Nziguheba H, Adam AM, Vanlauwe B (2021). Understanding nutrient imbalances in maize (*Zea mays* L.) using the diagnosis and recommendation integrated system (DRIS) approach in the Maize belt of Nigeria. Scientific Reports.

[ref-3] Ayamba BE, Abaidoo RC, Opoku A, Ewusi-Mensah N (2021). Enhancing the fertilizer value of cattle manure using organic resources for soil fertility improvement: a review. Journal of Bioresource Management.

[ref-4] Badu M (2014). Evaluation of interactive effects from combined cattle manure and mineral fertilizer application in sole maize cropping system. Master’s thesis.

[ref-5] Barłóg P, Hlisnikovský L, Kunzová E (2020). Effect of digestate on soil organic carbon and plant-available nutrient content compared to cattle slurry and mineral fertilization. Agronomy.

[ref-6] Blesh J, Hoey L, Jones AD, Friedmann H, Perfecto I (2019). Development pathways toward zero hunger. World Development.

[ref-7] Cardinael R, Eglin T, Guenet B, Neill C, Houot S, Chenu C (2015). Is priming effect a significant process for long-term SOC dynamics? Analysis of a 52-years old experiment. Biogeochemistry.

[ref-8] Chivenge P, Vanlauwe B, Gentile R, Wangechi H, Mugendi D, Van Kessel C, Six J (2009). Organic and mineral input management to enhance crop productivity in Central Kenya. Agronomy Journal.

[ref-9] Corn Growers’ Workshop (2018). Growth potential. https://www.pioneer.com/CMRoot/International/Australia_Intl/Publications/Corn_Workshop_Book.pdf.

[ref-10] Crews TE, Peoples MB (2004). Legume versus fertilizer sources of nitrogen: ecological tradeoffs and human needs. Agriculture, Ecosystems and Environment.

[ref-11] Crews TE, Peoples MB (2005). Can the synchrony of nitrogen supply and crop demand be improved in legume and fertilizer-based agroecosystems? A review. Nutrient Cycling in Agroecosystems.

[ref-12] Deligios PA, Farina R, Tiloca MT, Francaviglia R, Ledda L (2021). C-sequestration and resilience to climate change of globe artichoke cropping systems depend on crop residues management. Agronomy for Sustainable Development.

[ref-13] Deligios PA, Tiloca MT, Sulas L, Buffa M, Caraffini S, Doro L, Sanna G, Spanu E, Spissu E, Urracci GR, Ledda L (2017). Stable nutrient flows in sustainable and alternative cropping systems of globe artichoke. Agronomy for Sustainable Development.

[ref-14] Essel B, Abaidoo RC, Opoku A, Ewusi-Mensah N (2021). Mechanisms underlying nutrient interaction of compost and mineral fertilizer application in maize (*Zea mays* L.) cropping system in Ghana. Frontiers in Soil Science.

[ref-15] International Fertilizer Industry Association (IFA) (1992). Nutrient management guidelines for some major field crops.

[ref-16] Järvan M (2004). Available plant nutrients in growth substrate depending on various lime materials used for neutralizing bog peat. Agronomy Research.

[ref-17] Ji L, Wu Z, You Z, Yi X, Ni K, Guo S, Ruan J (2018). Effects of organic substitution for synthetic N fertilizer on soil bacterial diversity and community composition: a 10-year field trial in a tea plantation. Agriculture, Ecosystems & Environment.

[ref-18] Khan KS, Kunz R, Kleijnen J, Antea G (2003). Five steps to conducting a systematic review. Journal of the Royal Society of Medicine.

[ref-19] Khanal U, Wilson C, Rahman S, Lee BL, Hoang VN (2021). Smallholder farmers’ adaptation to climate change and its potential contribution to UN’s sustainable development goals of zero hunger and no poverty. Journal of Cleaner Production.

[ref-20] Kuzyakov Y (2010). Priming effects: interactions between living and dead organic matter. Soil Biology and Biochemistry.

[ref-21] Kuzyakov Y, Friedel JK, Stahr K (2000). Review of mechanisms and quantification of priming effects. Soil Biology and Biochemistry.

[ref-22] Lai CH, Settinayake ARH, Yeo WS, Lau SW, Jang TK (2019). Crop nutrients review and the impact of fertilizer of the plantation in Malaysia: a mini-review. Communications in Soil Science and Plant Analysis.

[ref-23] Liza MMJ, Islam MR, Jahiruddin M, Hasan MM, Alam MA, Shamsuzzaman SM, Samsuri AW (2014). Residual effects of organic manures with different levels of chemical fertilizers on rice. Life Science Journal.

[ref-24] Mahajan A, Gupta RD (2009). Integrated nutrient management (INM) in a sustainable rice-wheat cropping system.

[ref-25] Maize Production Manual (1982). (1) Manual Series (8).

[ref-26] Malvi UR (2011). Interaction of micronutrients with major nutrients with special reference to potassium. Karnataka Journal of Agricultural Sciences.

[ref-27] Mucheru M, Mugendi D, Micheni A, Mugwe J, Kung’u J, Otor S, Gitari J (2004). Improved food production by use of soil fertility amendment strategies in the central highlands of Kenya. Managing nutrient cycles to sustain soil fertility in Sub-Saharan Africa.

[ref-28] Nhamo N (2001). An evaluation of the efficacy of organic and inorganic fertilizer combinations in supplying nitrogen to crops. PhD thesis.

[ref-29] Oenema O, Velthof GL, Steenvoorden J, Claessen F, Willems J (2002). Balanced fertilization and regulating nutrient losses from agriculture. Agricultural effects on ground and surface waters: research at the edge of science and society.

[ref-30] Osemwota IO, Omueti JA, Ogboghodo AI (2007). Effect of calcium/magnesium ratio in soil on magnesium availability, yield, and yield components of maize. Communications in Soil Science and Plant Analysis.

[ref-31] Palm CA, Myers RJ, Nandwa SM (1997). Combined use of organic and inorganic nutrient sources for soil fertility maintenance and replenishment. Replenishing soil fertility in Africa.

[ref-32] Philips L (2021). Managing soil mineral ratios. Farmer’s Weekly. https://www.farmersweekly.co.za/crops/field-crops/managing-soil-mineral-ratios.

[ref-33] Rietra RP, Heinen M, Dimkpa CO, Bindraban PS (2017). Effects of nutrient antagonism and synergism on yield and fertilizer use efficiency. Communications in Soil Science and Plant Analysis.

[ref-34] Sait G (2015a). Six secrets to soil test success (Part 1). Nutrition Matters. http://blog.nutri-tech.com.au/six-secrets-to-soil-test-success-1/.

[ref-35] Sait G (2015b). Six secrets to soil test success (Part 2). Nutrition Matters. http://blog.nutri-tech.com.au/six-secrets-to-soil-test-success-2/.

[ref-36] Sanchez PA (2002). Soil fertility and hunger in Africa. Science.

[ref-37] Sanginga N, Woomer PL (2009). Integrated soil fertility management in Africa: principles, practices, and developmental process.

[ref-38] Scotti R, Bonanomi G, Scelza R, Zoina A, Rao MA (2015). Organic amendments as sustainable tool to recovery fertility in intensive agricultural systems. Journal of Soil Science and Plant Nutrition.

[ref-39] Tetteh FKM (2004). Synchronizing nutrient release from decomposing organic materials with crop nutrient demand in the semi-deciduous forest zone of Ghana. Doctoral dissertation.

[ref-40] Vanlauwe B, Aihou K, Aman S, Iwuafor ENO, Tossah BK, Diels J, Sanginga N, Mercks R, Deckers J (2001b). Maize yield as affected by organic inputs and urea in the West African moist savannah. Agronomy Journal.

[ref-41] Vanlauwe B, Palm C, Murwira H, Merckx R (2002). Organic resource management in sub-Saharan Africa: validation of a residue quality-driven decision support system. Agronomie.

[ref-42] Vanlauwe B, Sanginga N, Delve RJ, Probert ME (2004). The multiple roles of organic resources in implementing integrated soil fertility management strategies. Modelling nutrient management in tropical cropping systems. ACIAR proceedings no. 114.

[ref-43] Vanlauwe B, Wendt J, Diels J, Tian G, Ishida F, Keating JDH (2001a). Combined application of organic matter and fertilizer. Sustaining soil fertility in West Africa.

[ref-44] Wang JL, Liu KL, Zhao XQ, Zhang HQ, Li D, Li JJ, Shen RF (2021). Balanced fertilization over four decades has sustained soil microbial communities and improved soil fertility and rice productivity in red paddy soil. Science of The Total Environment.

[ref-45] Wu L, Zhang W, Wei W, He Z, Kuzyakov Y, Bol R, Hu R (2019). Soil organic matter priming and carbon balance after straw addition is regulated by long-term fertilization. Soil Biology and Biochemistry.

[ref-46] Yousaf M, Li J, Lu J, Ren T, Cong R, Fahad S, Li X (2017). Effects of fertilization on crop production and nutrient-supplying capacity under rice-oilseed rape rotation system. Scientific Reports.

