# Supplementary material for: Mechanisms for nutrient interactions from organic amendments and mineral fertilizer inputs under cropping systems: a review
Source: PeerJ. 2023 Apr 4;11:e15135. doi: 10.7717/peerj.15135 (PMC10081454; doi:10.7717/peerj.15135)
Supplement: Supplemental Information 1 [file peerj-11-15135-s001.pdf]

Search Methodology

This review began with the formulation of research questions and hypothesis, after which the scope was defined. The formulated research hypothesis was that "the nutrient interactions resulting from integrated application of organic amendments and mineral fertilizers to crops are influenced by mechanisms such as nutrient priming, nutrient synchrony, soil fertility and balanced ratio of nutrients". To ensure a rigorous investigation of literature so as to test the hypothesis and achieve the objectives of this review, a comprehensive investigation of published research on "mechanisms for nutrient interactions from organic amendments and mineral fertilizer inputs under cropping systems" was employed, following the approach by Khan *et al*. (2003). We conducted a literature search using Google scholar (scholar.google.com) and Scopus (Elsevier) literature database (https://www.scopus.com). Keywords and phrases such as "mechanisms for crop nutrient interactions", "nutrient priming effect", "nutrient synchrony", "soil fertility improvement", and "soil nutrient ratios" were used, while related articles were extracted to categorize and summarize the underlying mechanisms causing nutrient interactions from organic amendments and mineral fertilizer inputs under cropping systems. The search was restricted to references that included plant science and agronomy.
